# The Pre-Operative Duration of Symptoms: The Most Important Predictor of Post-Operative Efficacy in Patients with Degenerative Cervical Myelopathy

**DOI:** 10.3390/brainsci12081088

**Published:** 2022-08-17

**Authors:** Shengyu Guo, Taotao Lin, Rongcan Wu, Zhenyu Wang, Gang Chen, Wenge Liu

**Affiliations:** Department of Orthopedics, Fujian Medical University Union Hospital, Fujian Medical University, Fuzhou 350001, China

**Keywords:** degenerative cervical myelopathy, pre-operative duration of symptoms, post-operative efficacy, mJOA, mJOA recovery rate

## Abstract

**Objective.** To explore the most important predictors of post-operative efficacy in patients with degenerative cervical myelopathy (DCM). **Methods.** From January 2013 to January 2019, 284 patients with DCM were enrolled. They were categorized based on the different surgical methods used: single anterior cervical decompression and fusion (ACDF) (*n* = 80), double ACDF (*n* = 56), three ACDF (*n* = 13), anterior cervical corpectomy and fusion (ACCF) (*n* = 63), anterior cervical hybrid decompression and fusion (ACHDF) (*n* = 25), laminoplasty (*n* = 38) and laminectomy and fusion (*n* = 9). The follow-up time was 2 years. The patients were divided into two groups based on the mJOA recovery rate at the last follow-up: Group A (the excellent improvement group, mJOA recovery rate >50%, *n* = 213) and Group B (the poor improvement group, mJOA recovery rate ≤50%, *n* = 71). The evaluated data included age, gender, BMI, duration of symptoms (months), smoking, drinking, number of lesion segments, surgical methods, surgical time, blood loss, the Charlson Comorbidity Index (CCI), CCI classification, imaging parameters (CL, T1S, C2-7SVA, CL (F), T1S (F), C2-7SVA (F), CL (E), T1S (E), C2-7SVA (E), CL (ROM), T1S (ROM) and C2-7SVA (ROM)), maximum spinal cord compression (MSCC), maximum canal compromise (MCC), Transverse area (TA), Transverse area ratio (TAR), compression ratio (CR) and the Coefficient compression ratio (CCR). The visual analog score (VAS), neck disability index (NDI), modified Japanese Orthopedic Association (mJOA) and mJOA recovery rate were used to assess cervical spinal function and quality of life. **Results.** We found that there was no significant difference in the baseline data among the different surgical groups and that there were only significant differences in the number of lesion segments, C2–7SVA, T1S (F), T1S (ROM), TA, CR, surgical time and blood loss. Therefore, there was comparability of the post-operative recovery among the different surgical groups, and we found that there were significant differences in age, the duration of symptoms, CL and pre-mJOA between Group A and Group B. A binary logistic regression analysis showed that the duration of the symptoms was an independent risk factor for post-operative efficacy in patients with DCM. Meanwhile, when the duration of symptoms was ≥6.5 months, the prognosis of patients was more likely to be poor, and the probability of a poor prognosis increased by 0.196 times for each additional month of symptom duration (*p* < 0.001, OR = 1.196). **Conclusion.** For patients with DCM (regardless of the number of lesion segments and the proposed surgical methods), the duration of symptoms was an independent risk factor for the post-operative efficacy. When the duration of symptoms was ≥6.5 months, the prognosis of patients was more likely to be poor, and the probability of a poor prognosis increased by 0.196 times for each additional month of symptom duration (*p* < 0.001, OR = 1.196).

## 1. Introduction

Degenerative cervical myelopathy (DCM) is caused by cervical compression (at locations such as the vertebral bodies, intervertebral discs, ligaments and facet joints) and can be induced by cervical spinal cord compression. DCM can lead to functional nerve damage, which manifests as limb and trunk sensory impairment and motor dysfunction. The natural history of DCM includes a progressive worsening of signs and symptoms over time, but the rate and pattern of decline are unclear [1].

Recently, an increasing number of studies have compared the cervical sagittal parameters [2,3,4]. Xu Y [2] found that an increase in the T1S and NT and a decrease in the CL were risk factors affecting the post-operative NDI score. Kato M [3] found that after a laminoplasty, patients with C2-7SVA ≥ 35 mm will have a poor quality of life and severe neck pain. Nicholson KJ [4] found that greater C2-7ROM and an increased CL (F) corresponded to milder myelopathy symptoms. However, it has been reported that 1/3 of people with cervical kyphosis have no clinical symptoms [5,6]. Therefore, in post-operative efficacy research, we should not only consider the cervical sagittal parameters but should also pay attention to the natural history of DCM.

Magnetic resonance imaging (MRI) has become the imaging study of choice as the initial screening process for patients in whom DCM is suspected [7]. The maximum spinal cord compression (MSCC), maximum canal compromise (MCC), Transverse area (TA) and compression ratio (CR) have been widely used to evaluate the degree of spinal cord compression [8]. However, we must also recognize that there are some patients that have imaging manifestations of spinal cord compression but have no clinical symptoms [9,10].

Behrbalk E11 reported that the mean time delay from the initiation of symptoms to the diagnosis of DCM was 2.2 ± 2.3 years. What worries us is that this will lead to the prolongation of the natural history of DCM patients. Therefore, the purpose of this article is to explore the most important predictors of the post-operative efficacy in patients with degenerative cervical myelopathy by integrating the natural history of DCM, imaging parameters, number of lesion segments and different surgical methods.

## 2. Materials and Methods

### 2.1. Study Participants

**Figure 1:**
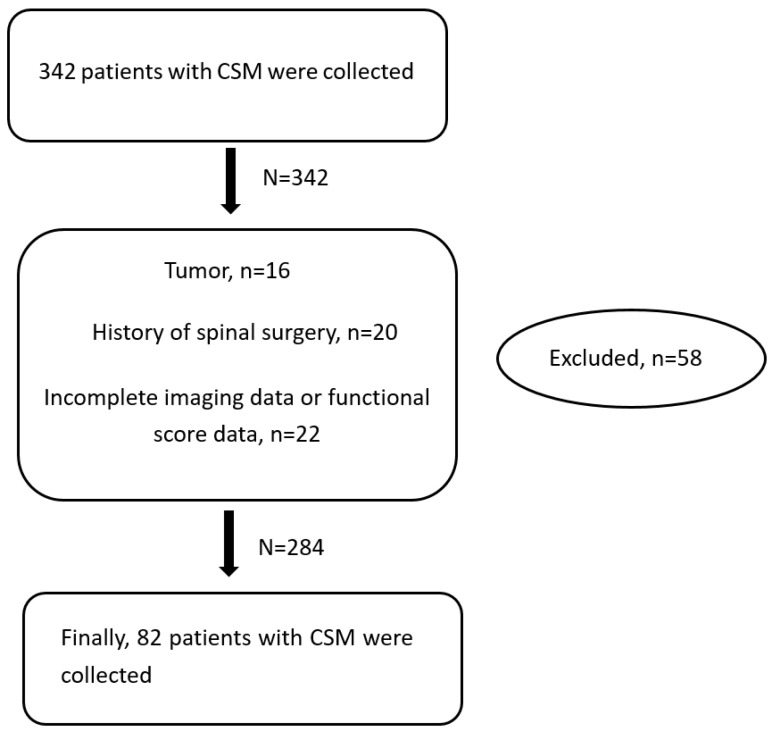
The summary of the details of patients excluded by inclusion criteria.

This study was a retrospective study, and 284 patients with DCM were enrolled from January 2013 to January 2019. Based on the surgical methods, the patients were divided into single ACDF (*n* = 80), double ACDF (*n* = 56), three ACDF (*n* = 13), ACCF (*n* = 63), ACHDF (*n* = 25), laminoplasty (*n* = 38) and laminectomy and fusion (*n* = 9) groups, and the follow-up time was 2 years. The inclusion criteria were as follows: (1) patients diagnosed with DCM based on their clinical symptoms and imaging data and who received surgical treatment in our hospital; (2) patients with complete and clear lateral cervical radiographs showing all the important bone markers that can be accurately measured; (3) patients with complete and clear cervical MR images that allow for the degree of cervical spinal cord compression to be accurately measured; and (4) patients with visual analog scale (VAS) results and modified Japanese Orthopedic Association (mJOA) and neck disability index (NDI) scores. The exclusion criteria were as follows: (1) a history of trauma or spinal surgery; (2) the presence of an infection, tuberculosis, a tumor or another disease; and (3) incomplete imaging data or functional score data. A summary of the details of the patients excluded by the inclusion criteria are shown in Figure 1. Finally, the patients were divided into two groups based on the mJOA recovery rate at the last follow-up: Group A (the excellent improvement group, mJOA recovery rate >50%, *n* = 213) and Group B (the poor improvement group, mJOA recovery rate ≤50%, *n* = 71). This study passed the ethical review of the ethics committee. The ethical review number is 2021KY143.

### 2.2. The Basic Data Collected and Parameters Measured

#### The Basic Data Included the Following

(1) Age, sex, BMI, duration of symptoms (months), smoking history, drinking history, number of lesion segments, surgical methods, surgical time (min) and blood loss (mL). (Smoking history: those who smoke more than one cigarette a day for more than 6 months or those who had smoked more than 100 cigarettes in total. Drinking history: those who drink at least once a week for more than half a year.)

**Figure 2:**
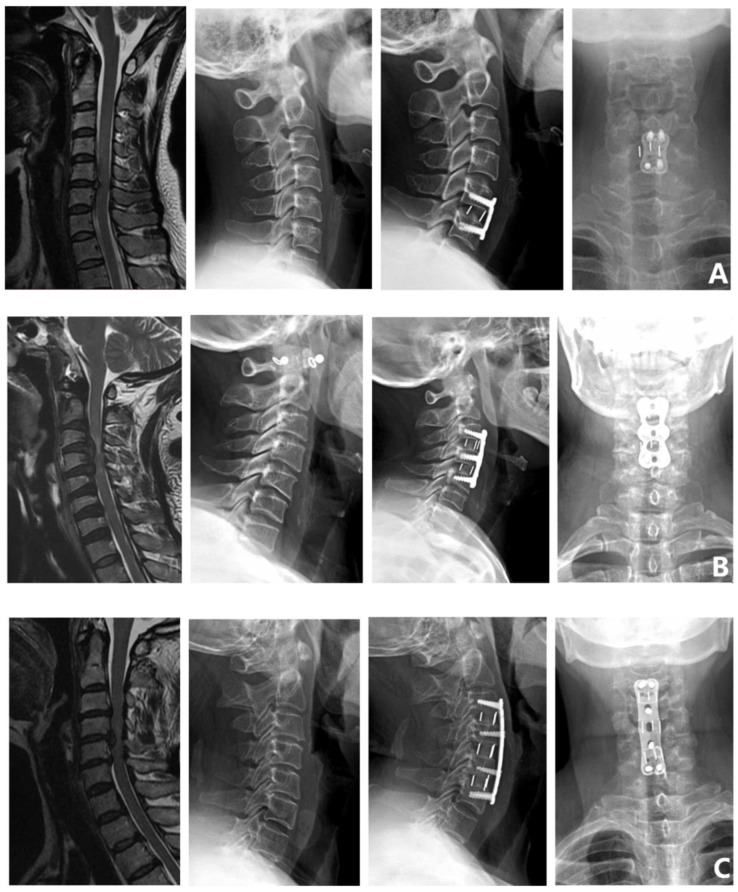

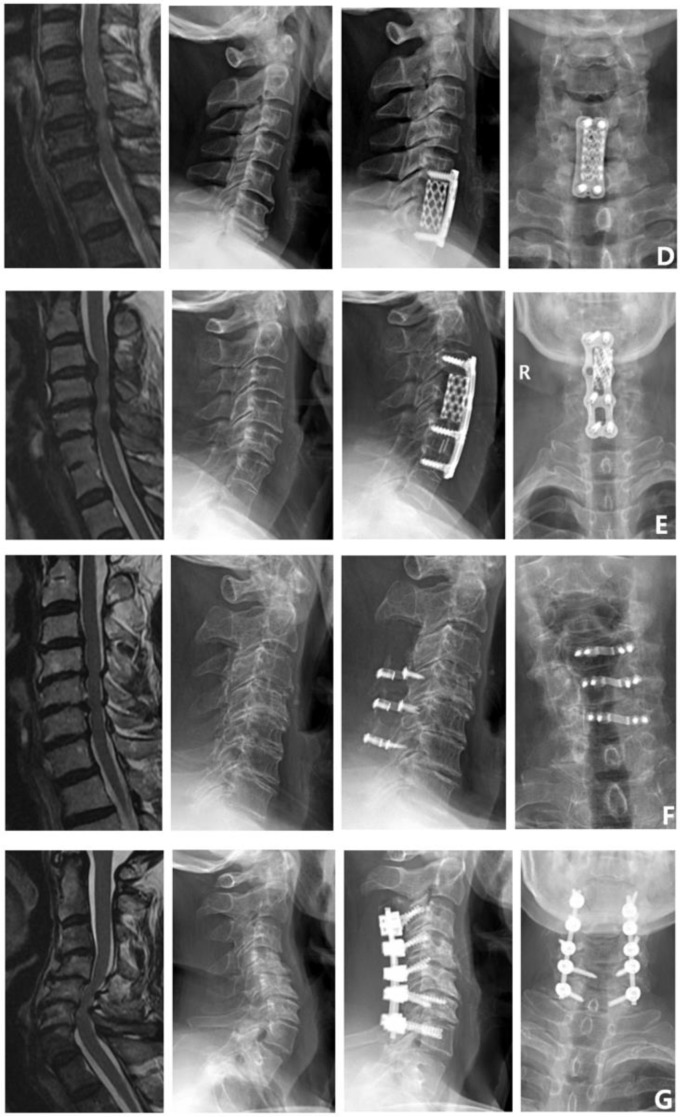
Surgical methods. (**A**) Single ACDF. (**B**) Double ACDF. (**C**) Three ACDF. (**D**) ACCF. (**E**) ACHDF. (**F**) Laminoplasty. (**G**) Laminectomy and fusion.

Surgical methods: ① Single-segment anterior cervical decompression and fusion (single ACDF). ② Double-segment anterior cervical decompression and fusion (double ACDF). ③ Three-segment anterior cervical decompression and fusion (three ACDF). ④ Anterior cervical corpectomy and fusion (ACCF). ⑤ Anterior cervical hybrid decompression and fusion (ACHDF). ⑥ Posterior cervical laminectomy (laminoplasty). ⑦ Posterior cervical laminectomy and fusion (laminectomy and fusion) (Figure 2).

**Figure 3:**
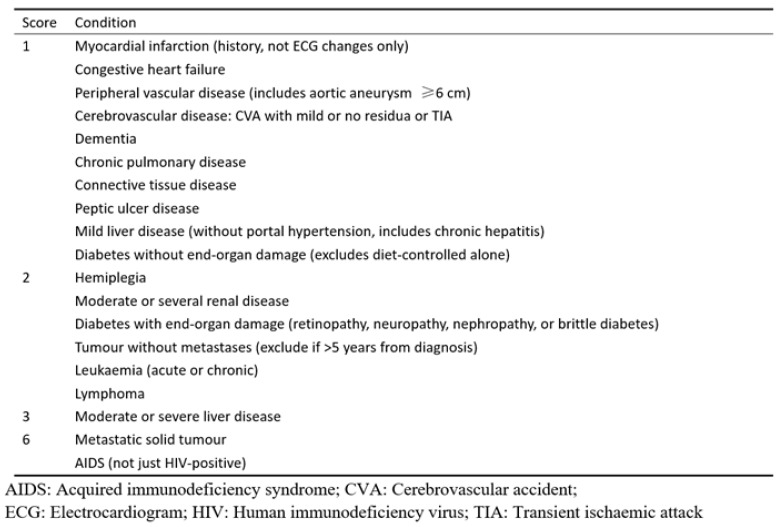
Charlson Comorbidity Index (CCI).

(2) The Charlson Comorbidity Index (CCI) and the CCI classification. This study used the Charlson Comorbidity Index (CCI) to assess the comorbidity data in DCM patients. The CCI score is based on a number of conditions, including previous myocardial infarction, stroke and liver disease, which are each assigned different weights, with a higher weight representing a more severe morbidity. The summation of the weighted comorbidity scores results in a summary score (Figure 3). For the statistical analysis, patients in this study were divided into two groups based on their CCI score: CCI 0–1 and CCI ≥ 2.

(3) Imaging parameters. For the lateral cervical radiographs, standard radiographic techniques were applied. The tube-to-subject distance was 1.83 m and the radiographic tube was centered at the C4-C5 intervertebral disc space without magnification. Lateral radiographs of the cervical spine were taken when the patient was in a comfortable standing position; the upper extremities were positioned naturally at the sides of the trunk and the patient looked straight ahead.

**Figure 4:**
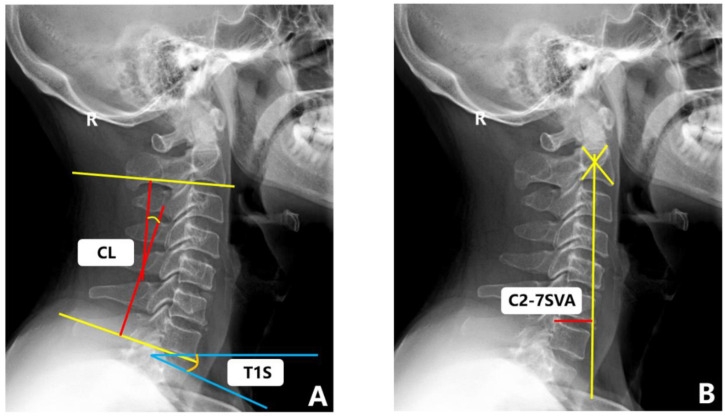
Sagittal radiograph of cervical spine with representative measurements. (**A**) C2-C7 cobb angle (CL), T1 slope (T1S). (**B**) C2-7SVA.

Flexion (or extension) cervical radiograph. The patient stood sideways in front of the camera frame, the head and neck were maximally flexed (or extended), the long axis of the neck was parallel to the long axis of the film, and the shoulders were drooped as far as possible. The remaining requirements were the same as those of the lateral films. All of the above images were captured by the same imaging technician. The measurement methods of the cervical sagittal parameters in the radiographs are detailed in Table 1 and Figure 4. The range of motion (ROM) was calculated as the extension minus the flexion.

**Figure 5:**
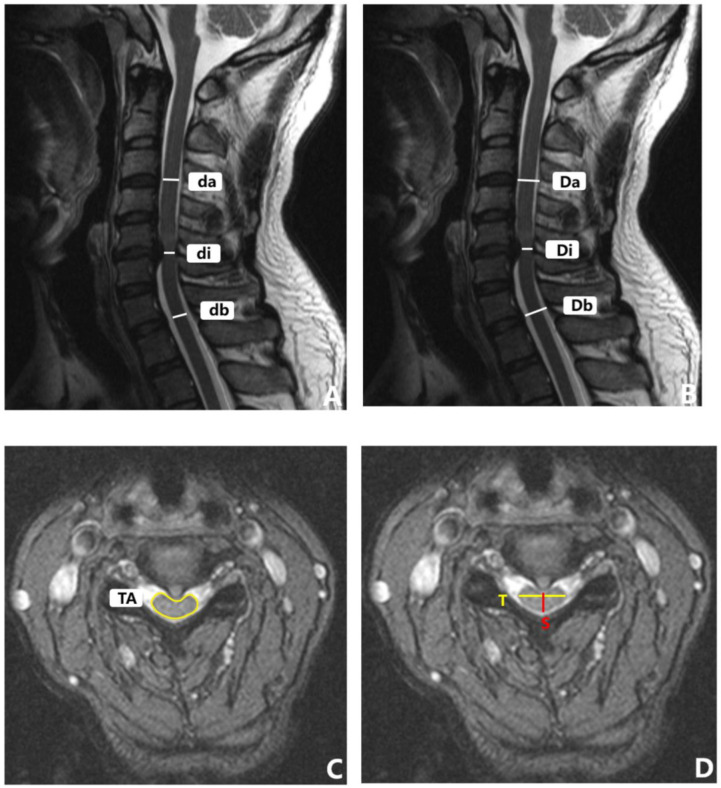
MRI parameter measurement of cervical spine. (**A**) MSCC. (**B**) MCC. (**C**) TA. (**D**) CR.

Every patient underwent a pre-operative 3.0 T MRI scan to assess the degree of spinal cord compression, and all the enrolled patients underwent a 3.0 T magnetic resonance imaging (MRI) scan (Siemens Medical Solutions, Erlangen, Germany) pre-operatively. The axial MRI images were aligned parallel to the inferior endplate of the vertebral body. The methods that were used to measure the cervical MRI parameters are detailed in Table 1 and Figure 5. For patients with multilevel DCM, we measured the segment with the most severe spinal cord compression.

### 2.3. Outcome Measures

(1) The visual analog score (VAS) was reported based on an 11-point numeric rating scale from zero (no pain) to ten (worst pain imaginable).

(2) The neck disability index (NDI) was used to evaluate neck function. The patients were evaluated based on their pain intensity, ability for self-care, weightlifting, reading ability, presence of headaches, concentration, work, sleep quality, driving and recreational activities. The total score was 50. The higher the score, the worse the neck function.

(3) The modified Japanese Orthopedic Association (mJOA) scale consisted of three categories: exercise, sensation and bladder function. The total score was 18 points.

(4) mJOA recovery rate = [(mJOA score after treatment − mJOA score before treatment)/(18 − mJOA score before treatment)] × 100%. In our study, the patients were divided into the excellent improvement group (Group A, mJOA recovery rate >50%, *n* = 213) and the poor improvement group (Group B, mJOA recovery rate ≤50%, *n* = 71) based on the mJOA recovery rate at the last follow-up.

#### 2.3.1. Statistical Analysis

All images were transferred to a computer as DICOM data. The measurements were performed with imaging software (OsiriX Lite v 7.5; Icestar Media Ltd., Essex, UK) by 2 independent observers. After an agreement was reached between the observers, each parameter was independently measured twice by 2 orthopedic spine surgeons, and the intraclass correlation coefficient (ICC) was analyzed. Intra- and interobserver agreement were assessed via the ICC, and ICC values of 0.8 to 1.0, 0.6 to 0.79 and less than 0.6 were considered excellent, good and poor, respectively.

SPSS 24.0 was used for the statistical analyses, and statistically significant differences were identified when the *p*-value was <0.05. For continuous variables, the Shapiro–Wilk normal test was used; normally distributed continuous variables were expressed in the form of Mean ± Standard Deviation and nonnormally distributed continuous variables were expressed in the form of the Median (Lower quartile~Upper quartile). The intergroup analysis of the different surgical methods was performed as follows: for categorical variables, the chi-square test was used; for normally distributed continuous variables, a one-way ANOVA was used; and for nonnormally distributed continuous variables, the Kruskal–Wallis H test was used. The intergroup analysis of Group A and Group B was performed as follows: for categorical variables, the chi-square test was used; for normally distributed continuous variables, an independent-sample *t*-test was used; and for nonnormally distributed continuous variables, the Mann–Whitney U test was used. Binary logistic regression analysis and ROC curves were used to determine the independent risk factors and critical values, respectively. Pearson and Spearman correlation coefficients were used to calculate the correlation between each parameter.

#### 2.3.2. Reliability Analysis

Regarding the cervical spine parameters, the intraobserver and interobserver reliability results showed excellent and good agreement, respectively (ICC ≥ 0.8).

## 3. Results

### 3.1. Basic Data and the Comparison among the Different Surgical Method Groups

From January 2013 to January 2019, a total of 284 patients with DCM were enrolled. The patients were divided based on the different surgical methods into a single ACDF (*n* = 80), double ACDF (*n* = 56), three ACDF (*n* = 13), ACCF (*n* = 63), ACHDF (*n* = 25), laminoplasty (*n* = 38) or laminectomy and fusion (*n* = 9) group. The follow-up time was 2 years (Table 2).

In the statistical analysis, we found that there was no significant difference in the baseline data, such as in age, sex, BMI, duration of symptoms, severity of pre-operative symptoms and post-operative recovery or among the different surgical groups (*p* > 0.05); there were only significant differences in the number of lesion segments, C2–7SVA, T1S (F), T1S (ROM), TA, CR, surgical time and blood loss. Therefore, in exploring the differences in post-operative recovery, there is comparability among the different surgical groups (Table 2).

### 3.2. Comparison between the Excellent Improvement Group and Poor Improvement Group

The patients were divided into two groups based on the mJOA recovery rate at the last follow-up: Group A (the excellent improvement group, mJOA recovery rate >50%, *n* = 213) and Group B (the poor improvement group, mJOA recovery rate ≤50%, *n* = 71) (Table 3).

The excellent improvement group (Group A) included 213 patients, 138 males (64.8%) and 75 females (35.2%), with an average age of 54.3 ± 10.8 years. The mean BMI was 23.8 ± 3.0 kg/m^2^ and the mean duration of symptoms was 2.0 (1.0~5.0) months. The details are described in Table 3.

The poor improvement group (Group B) included 71 patients, 43 males (60.6%) and 28 females (39.4%), with an average age of 57.6 ± 10.4 years. The mean BMI was 23.5 ± 2.5 kg/m^2^ and the mean duration of symptoms was 24.0 (12.0~36.0) months. The details are described in Table 3.

**Figure 6:**
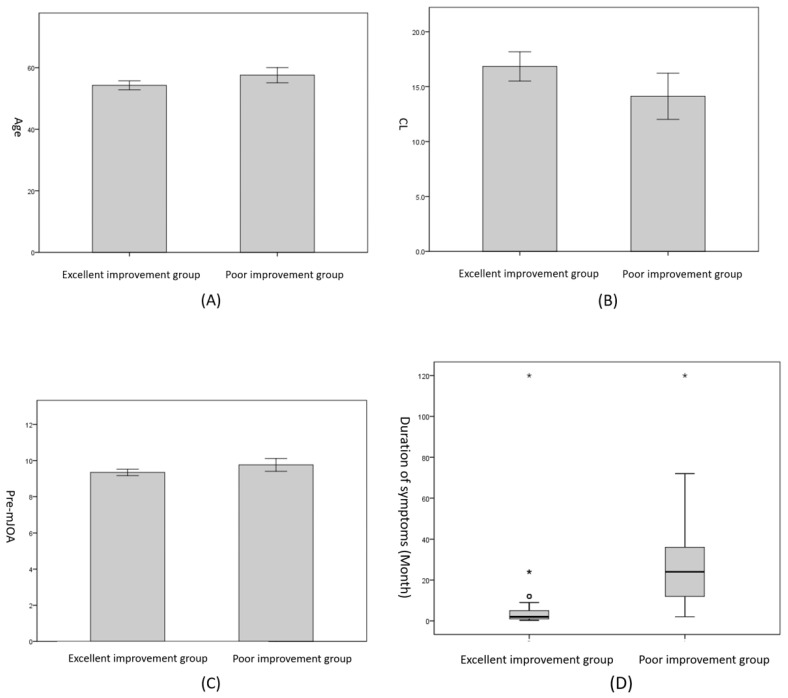
(**A**) Age difference between the two groups (independent-sample *t*-test). (**B**) CL difference between the two groups (independent-sample *t*-test). (**C**) Pre-mJOA difference between the two groups (independent-sample *t*-test). (**D**) Duration of symptoms (months) difference between the two groups (Mann–Whitney U test). Asterisks represent data with large bias.

There was no significant difference between Group A and Group B regarding gender, BMI, smoking history, drinking history, number of lesion segments, surgical methods, surgical time, blood loss, the CCI, CCI classification, T1S, C2–7SVA, CL (F), T1S (F), C2–7SVA (F), CL (E), T1S (E), C2–7SVA (E), CL (ROM), T1S (ROM), C2–7SVA (ROM), MSCC, MCC, TA, TAR, CR, CCR, pre-VAS and pre-NDI (*p* > 0.05). There were significant differences in age, duration of symptoms, the CL and the pre-mJOA (*p* < 0.05) (Table 3 and Figure 6).

**Figure 7:**
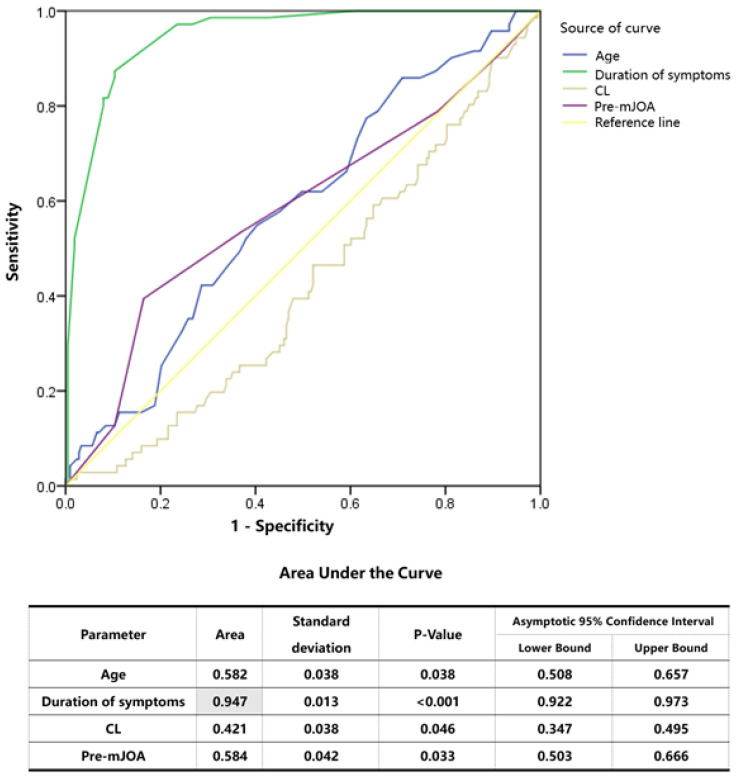
ROC curve (age, duration of symptoms, CL and pre-mJOA).

After a binary logistic regression analysis, we found that only the duration of symptoms was an independent risk factor for the post-operative efficacy in patients with DCM (*p* < 0.001) (Table 4). To further judge the influencing degree of each risk factor, we used ROC curves for comparative judgment. Based on the ROC curve, the prediction accuracy of the duration of symptoms was the highest (the area under the curve was 0.947) (Figure 7). Through the calculation of the critical values, we found that when the duration of symptoms was ≥ 6.5 months, the prognosis of patients was more likely to be poor, and the probability of a poor prognosis increased by 0.196 times for each additional month of symptom duration (*p* < 0.001, OR = 1.196).

### 3.3. Correlation between the Pre-operative Basic Data, Imaging Parameters and Post-Operative Efficacy

Pearson and Spearman correlation coefficients were used to calculate the correlation between the pre-operative basic data, imaging parameters and post-operative efficacy. We found that age was negatively correlated with the post-mJOA and mJOA recovery rates and that the duration of symptoms was positively correlated with the post-VAS and post-NDI scores and was negatively correlated with the post-mJOA and mJOA recovery rates. The CL was positively correlated with the post-mJOA and mJOA recovery rates. The MSCC, MCC, TAR and CCR were significantly negatively correlated with the pre-mJOA. The TA and CR were significantly positively correlated with the pre-mJOA. The details are described in Table 5.

## 4. Discussion

Degenerative cervical myelopathy will lead to progressive spinal cord injury and can cause serious physical and social disability, which will cause a heavy economic burden for patients and society [11]. As a common method for the treatment of DCM patients, surgery has been widely used in the clinic. The surgery includes completely decompressing the compressed spinal cord and reconstructing the structural stability of the cervical spine to create favorable conditions for the recovery of spinal cord function and the improvement of clinical symptoms. The common methods of anterior cervical surgery are ACDF, ACCF and ACHDF. The common methods of posterior cervical surgery are laminoplasty, laminectomy and fusion. Many factors should be considered when deciding on the surgical approach and mode for patients with DCM, and the factors to consider include the clinical symptoms, signs, imaging findings (cervical spinal cord compression factors and responsible segments, degeneration range, cervical curvature and stability), possible surgical complications, operative habits of the surgeons, medical expenses, etc. [12,13,14]. In our study, 284 patients with DCM treated with different surgical methods were enrolled. After comparing the baseline data, we found that there was no significant difference in the basic data, the severity of the pre-operative symptoms or the post-operative recovery; therefore, it is important to explore the risk factors that affect the post-operative recovery. This study demonstrates that for patients with DCM, the curative effect of surgery is accurate and reliable regardless of which surgical method is adopted, as long as the compressed segment of the spinal cord can be completely decompressed and the structural stability of the cervical spine can be reconstructed.

The cervical sagittal parameters and the degree of spinal cord compression, as important evaluation indexes, are widely used to evaluate the severity of symptoms and the post-operative efficacy in patients with DCM [15,16]. In our study, we found that the CL, CL (F), MSCC, MCC, TA, TAR, CR and CCR were all related to the severity of the pre-operative symptoms, which was consistent with the findings of the above scholars. In the study of risk factors affecting the post-operative efficacy, we found that the older the age of the patient, the longer the duration of the symptoms are, and we also found that a smaller CL and a worse neurological functional score was related to a worse post-operative efficacy.

The natural history of DCM includes a progressive worsening of signs and symptoms over time, but the rate and pattern of decline are unclear [1]. Two main patterns of DCM progression have been reported: (1) a slow worsening of function over time and (2) an extended period of stable neurological function followed by expedited decline [17]. Therefore, the natural history of DCM has an important relationship with the prognosis of patients. In our study, we found that only the duration of symptoms was an independent risk factor for the post-operative efficacy in patients with DCM (*p* < 0.001) after a binary logistic regression analysis. To further judge the influencing degree of each risk factor, we used ROC curves for comparative judgment. Based on the ROC curve, the prediction accuracy for the duration of symptoms was the highest (the area under the curve was 0.947). After the calculation of critical values, we found that when the duration of symptoms was ≥6.5 months, the prognosis of patients was more likely to be poor, and the probability of a poor prognosis increased by 0.196 times for each additional month of symptom duration (*p* < 0.001, OR = 1.196). Behrbalk E [18] reported that the mean time delay from the initiation of symptoms to the diagnosis of DCM was 2.2 ± 2.3 years. What worries us is that this will lead to the prolongation of the natural history of DCM patients. Therefore, attention to the natural history of patients with DCM is critical.

Since the Charlson Comorbidity Index (CCI) was proposed, an increasing number of studies have shown that it has a significant correlation with the mortality, prognosis and curative effect [19,20,21]. However, the application of the CCI is still limited in the field of spinal surgery. Sim DS [21] divided the patients into two groups by the CCI (CCI 0–1 and CCI ≥ 2) and found that the CCI was an independent risk factor for the Parker mobility score after hip fracture surgery. Similarly, we also adopted this grouping, but we did not find a correlation between the CCI and the prognosis of patients with DCM. This may be because the main source of symptoms in patients with DCM is spinal cord compression, while the symptoms caused by comorbidities are less related to the spinal cord compression.

However, our research also has the following shortcomings and issues that need to be further explored. First, the number of cases in this study is limited. Second, this study was a retrospective, single-center study. Prospective and multicenter research is needed to further clarify the correlation between the duration of symptoms and the post-operative efficacy in patients with DCM.

## 5. Conclusions

For patients with DCM (regardless of the number of lesion segments and the proposed surgical methods), the duration of symptoms was an independent risk factor for the post-operative efficacy. When the duration of symptoms is ≥6.5 months, the prognosis of patients will be more likely to be poor, and the probability of a poor prognosis will increase by 0.196 times for each additional month of symptom duration (*p* < 0.001, OR = 1.196).

## Figures and Tables

**Table 1:** The measuring methods of cervical sagittal parameters in the radiographs and MSCC, MCC, TA, TAR, CR, CCR in MRI.

Cervical Sagittal Parameters	The Measuring Method (Figure 4)
C2-C7 cobb angle (CL)	The cobb angle between the C2 lower endplate and C7 lower endplate, with (+) for lordosis and (−) for kyphosis.
T1 slope (T1S)	The angle between a horizontal line and the T1 superior endplate.
C2-7 sagittal vertical axis (SVA)	The distance between the plumb line through the center of C2 and the plumb line of the posterior of the C7 upper endplate.
Parameters in MRI	The measuring method (Figure 5).
Maximum spinal cord compression (MSCC)	A midsagittal T2-WI of a patient with DCM, including the width of the spinal cord at the most compressed site (di) and the width of the spinal cord at normal sites above (da) and below (db) the site of maximum compression. MSCC = 1 − di/[(da + db)/2].
Maximum canal compromise (MCC)	A midsagittal T2-WI of a patient with DCM, including the width of the canal at the most stenotic site (Di) and the width of the spinal canal at normal sites above (Da) and below (Db) the canal stenosis. MCC = 1 − Di/[(Da + Db)/2].
Transverse area (TA)	A midsagittal T2-WI of a patient with DCM, the cross-sectional area of the spinal cord at the most severely compressed segment was measured.
Transverse area ratio (TAR)	A midsagittal T2-WI of a patient with DCM, including the cross-sectional area of compressed spinal cord (TA) and the cross-sectional area of upper and lower normal spinal cord (TA1,TA2). TAR = 1 − TA/[(TA1 + TA2)/2].
Compression ratio (CR)	A midsagittal T2-WI of a patient with DCM, a ratio between the anteroposterior diameter and the transverse diameter on the cross-sectional of spinal cord compression. CR = S/T.
Coefficient compression ratio (CCR)	A midsagittal T2-WI of a patient with DCM, including the CR of compressed spinal cord (CR1) and the CR of upper and lower normal spinal cord (CR2,CR3). CCR = 1 − CR1/[(CR2 + CR3)/2].

**Table 2:** Basic data and comparison among different surgical methods group.

	Single-ACDF (*N* = 80)	Double-ACDF (*N* = 56)	Three-ACDF (*N* = 13)	ACCF (*N* = 63)	ACHDF (*N* = 25)	Laminoplasty (*N* = 38)	Laminectomy and Fusion (*N* = 9)	× ^2^/F	*p*-Value
Age	53.1 ± 10.0	56.6 ± 11.9	50.1 ± 9.2	54.3 ± 11.5	57.6 ± 9.9	58.2 ± 10.3	56.6 ± 6.8	1.985	0.068
Gender (Female, *n* (%))	36 (45%)	24 (42.9%)	4 (30.8%)	23 (36.5%)	4 (16%)	9 (23.7%)	3 (33.3%)	10.942	0.09
BMI	24.1 ± 2.7	23.4 ± 3.2	24.1 ± 2.2	23.6 ± 3.1	23.2 ± 2.9	23.5 ± 3.0	24.3 ± 2.7	0.583	0.744
Duration of symptoms (months)	3.5 (2.0~8.0)	3.5 (1.0~12.0)	2.0 (1.0~6.0)	4.0 (1.0~12.0)	4.0 (1.5~12.0)	3.0 (1.0~12.0)	2.0 (1.5~7.5)	1.856	0.932
Smoking (*n* (%))	18 (22.5%)	19 (33.9%)	3 (23.1%)	21 (33.3%)	10 (40%)	14 (36.8%)	2 (22.2%)	5.363	0.498
Drinking (*n* (%))	23 (28.8%)	14 (25%)	3 (23.1%)	19 (30.2%)	9 (36%)	9 (23.7%)	3 (33.3%)	1.835	0.934
CCI	2.0 (0.0~3.0)	2.0 (1.0~3.0)	1.0 (0.0~2.5)	2.0 (0.0~3.0)	2.0 (0.5~3.5)	2.0 (0.0~3.0)	3.0 (1.5~3.5)	9.735	0.136
CCI classification (0–1 point, *n* (%))	36 (45%)	21 (37.5%)	8 (61.5%)	31 (49.2%)	8 (32%)	16 (42.1%)	2 (22.2%)	6.468	0.373
CL	16.9 ± 9.7	14.8 ± 10.1	14.4 ± 7.8	17.1 ± 9.5	17.5 ± 10.8	15.6 ± 9.9	12.8 ± 5.1	0.727	0.629
T1S	24.8 ± 8.2	24.6 ± 7.7	21.2 ± 4.8	26.8 ± 6.6	25.2 ± 7.5	25.5 ± 7.2	26.0 ± 5.7	1.222	0.295
C2-7SVA (mm)	16.0 ± 11.0	17.0 ± 12.1	14.7 ± 9.5	14.4 ± 10.9	15.0 ± 11.4	14.6 ± 10.7	28.4 ± 13.1	2.260	0.038 *
CL(F)	−18.9 ± 9.8	−17.4 ± 10.1	−17.8 ± 8.7	−15.9 ± 9.6	−18.4 ± 8.8	−19.3 ± 8.7	−15.7 ± 9.5	0.873	0.515
T1S(F)	32.2 ± 10.3	34.2 ± 10.0	28.2 ± 6.4	37.7 ± 7.7	36.3 ± 5.7	33.3 ± 7.2	31.1 ± 6.1	3.978	0.001 **
C2-7SVA (F)	62.3 ± 16.8	63.3 ± 13.4	53.6 ± 14.7	64.5 ± 11.6	65.8 ± 9.6	62.1 ± 11.0	63.0 ± 12.7	1.435	0.201
CL(E)	28.9 ± 11.9	27.1 ± 13.0	21.8 ± 8.8	28.1 ± 11.6	24.6 ± 12.0	25.9 ± 12.3	25.1 ± 10.2	1.083	0.373
T1S(E)	22.1 ± 9.8	22.3 ± 7.8	17.5 ± 6.2	23.0 ± 9.7	20.5 ± 8.8	22.4 ± 7.9	23.2 ± 5.2	0.867	0.519
C2-7SVA (E)	1.5 (−12.7~7.5)	3.7 (−5.9~8.9)	5.8 (−5.3~16.0)	2.0 (−15.8~6.4)	3.0 (−15.2~11.3)	−1.8 (−9.7~7.0)	7.5 (3.7~14.0)	11.447	0.075
CL(ROM)	47.8 ± 14.3	44.5 ± 13.2	39.5 ± 11.2	44.0 ± 13.6	43.0 ± 14.5	45.3 ± 13.1	40.8 ± 11.8	1.202	0.305
T1S(ROM)	−10.1 ± 9.0	−11.8 ± 10.2	−10.8 ± 7.1	−14.7 ± 12.1	−15.8 ± 9.5	−11.0 ± 8.2	−7.9 ± 6.2	2.323	0.033 *
C2-7SVA (ROM)	−63.1 ± 24.7	−60.8 ± 20.1	−49.1 ± 19.2	−68.1 ± 21.2	−67.2 ± 21.0	−63.2 ± 18.0	−54.8 ± 17.1	1.976	0.069
MSCC	0.39 ± 0.14	0.40 ± 0.11	0.44 ± 0.15	0.44 ± 0.12	0.45 ± 0.14	0.42 ± 0.11	0.46 ± 0.10	1.709	0.119
MCC	0.50 ± 0.15	0.52 ± 0.13	0.56 ± 0.11	0.56 ± 0.12	0.55 ± 0.15	0.52 ± 0.13	0.55 ± 0.14	1.420	0.207
TA	0.52 ± 0.15	0.48 ± 0.11	0.50 ± 0.08	0.44 ± 0.13	0.44 ± 0.15	0.45 ± 0.14	0.49 ± 0.22	2.748	0.013 *
TAR	0.40 ± 0.13	0.43 ± 0.12	0.43 ± 0.09	0.46 ± 0.12	0.45 ± 0.14	0.45 ± 0.11	0.48 ± 0.11	1.793	0.1
CR	0.26 ± 0.08	0.24 ± 0.07	0.22 ± 0.07	0.21 ± 0.08	0.21 ± 0.07	0.23 ± 0.09	0.22 ± 0.06	2.784	0.012 *
CCR	0.44 ± 0.15	0.46 ± 0.13	0.50 ± 0.14	0.51 ± 0.14	0.50 ± 0.16	0.49 ± 0.13	0.46 ± 0.13	1.843	0.091
Pre-VAS	4.1 ± 2.1	4.4 ± 2.3	4.1 ± 2.7	4.4 ± 2.4	5.0 ± 2.5	4.1 ± 2.5	4.7 ± 2.1	0.607	0.725
Pre-NDI	15.8 ± 8.3	17.1 ± 9.3	17.0 ± 10.8	17.8 ± 9.2	17.8 ± 9.1	13.9 ± 9.4	19.7 ± 7.8	1.127	0.347
Pre-mJOA	9.8 ± 1.5	9.6 ± 1.4	9.3 ± 0.9	9.3 ± 1.3	9.0 ± 1.3	9.3 ± 1.2	9.0 ± 1.6	1.680	0.126
Surgical time (min)	100.2 ± 14.9	133.5 ± 16.5	158.7 ± 13.7	118.1 ± 16.6	140.5 ± 18.4	139.4 ± 13.8	179.2 ± 19.5	73.238	<0.001 **
Blood loss (mL)	51.1 ± 16.9	74.7 ± 20.8	102.3 ± 10.7	192.7 ± 34.0	220.2 ± 36.8	174.5 ± 31.6	289.4 ± 46.9	320.761	<0.001 **
Post-VAS (1)	2.1 ± 0.9	2.1 ± 1.1	2.7 ± 0.9	2.1 ± 1.1	2.1 ± 1.2	2.1 ± 1.1	2.8 ± 0.8	1.181	0.316
Post-NDI (1)	8.0 ± 4.6	9.4 ± 5.1	9.9 ± 4.7	8.8 ± 5.2	9.5 ± 5.4	8.0 ± 5.4	9.3 ± 4.4	0.829	0.548
Post-mJOA (1)	15.6 ± 1.5	15.2 ± 1.8	16.2 ± 1.2	15.5 ± 1.9	14.9 ± 2.0	15.0 ± 2.4	15.1 ± 1.9	1.393	0.217
mJOA recovery rate% (1)	69.6 ± 19.9	65.4 ± 22.7	77.4 ± 17.7	71.6 ± 21.1	65.6 ± 20.5	64.9 ± 27.3	68.3 ± 20.0	1.041	0.399
Post-VAS (3)	1.8 ± 0.9	1.7 ± 0.9	1.5 ± 1.0	1.8 ± 0.9	1.9 ± 1.0	1.9 ± 0.9	1.9 ± 0.6	0.456	0.840
Post-NDI (3)	5.9 ± 4.3	7.1 ± 4.8	7.8 ± 3.8	7.0 ± 4.4	7.8 ± 4.4	5.9 ± 4.2	7.9 ± 4.1	1.280	0.266
Post-mJOA (3)	15.5 ± 1.5	15.2 ± 1.8	16.1 ± 1.0	15.6 ± 1.7	14.9 ± 2.0	15.0 ± 2.3	15.1 ± 1.9	1.293	0.260
mJOA recovery rate% (3)	68.9 ± 19.8	65.6 ± 22.4	76.6 ± 16.1	71.8 ± 20.0	65.7 ± 20.8	64.9 ± 26.4	68.3 ± 20.0	0.981	0.438
Post-VAS (6)	1.6 ± 0.9	1.6 ± 0.8	1.3 ± 0.6	1.7 ± 0.8	1.7 ± 0.7	1.7 ± 0.8	1.7 ± 0.5	0.648	0.692
Post-NDI (6)	5.7 ± 4.7	6.2 ± 4.3	6.5 ± 2.4	6.4 ± 4.2	6.8 ± 3.2	5.8 ± 4.3	7.8 ± 3.1	0.634	0.703
Post-mJOA (6)	15.5 ± 1.5	15.1 ± 1.8	15.9 ± 1.0	15.5 ± 1.8	14.9 ± 2.0	15.0 ± 2.3	15.0 ± 2.1	1.064	0.384
mJOA recovery rate% (6)	67.9 ± 20.0	64.6 ± 22.2	74.9 ± 15.2	70.9 ± 20.1	65.7 ± 20.8	64.6 ± 26.9	67.2 ± 21.4	0.830	0.548
Post-VAS (12)	1.7 ± 1.0	1.5 ± 0.7	1.3 ± 0.6	1.7 ± 0.8	1.8 ± 0.8	1.7 ± 0.8	1.7 ± 0.5	0.776	0.589
Post-NDI (12)	5.5 ± 4.3	5.6 ± 3.7	6.4 ± 1.7	6.3 ± 4.0	6.1 ± 4.6	5.6 ± 4.1	7.6 ± 2.8	0.582	0.745
Post-mJOA (12)	15.4 ± 1.5	15.0 ± 1.9	15.8 ± 1.0	15.4 ± 1.8	14.8 ± 1.9	14.7 ± 2.3	15.0 ± 2.1	1.124	0.348
mJOA recovery rate% (12)	66.8 ± 20.2	63.1 ± 23.4	73.2 ± 15.4	69.4 ± 20.6	64.8 ± 20.0	62.3 ± 26.7	67.2 ± 21.4	0.876	0.513
Post-VAS (24)	1.8 ± 1.0	1.6 ± 0.8	1.3 ± 0.6	1.8 ± 0.8	2.1 ± 1.1	1.8 ± 0.8	1.8 ± 0.7	1.692	0.123
Post-NDI (24)	6.0 ± 4.8	7.7 ± 5.1	6.3 ± 1.1	6.5 ± 4.3	7.8 ± 6.0	5.9 ± 4.1	8.0 ± 2.0	1.306	0.255
Post-mJOA (24)	15.1 ± 1.6	14.6 ± 1.9	15.4 ± 1.0	15.1 ± 1.9	14.3 ± 2.2	14.5 ± 2.3	14.2 ± 1.9	1.475	0.187
mJOA recovery rate% (24)	63.0 ± 20.8	59.0 ± 24.6	68.4 ± 17.9	66.8 ± 21.0	58.9 ± 23.0	59.1 ± 26.1	58.1 ± 19.3	1.081	0.374

* and lighter gray shading signifies that *p* < 0.05. ** and dark gray shading signifies that *p* < 0.01.

**Table 3:** Basic data and comparison between Group A and Group B.

	Group A (Excellent Improvement) (*n* = 213)	Group B (Poor Improvement) (*n* = 71)	T/× ^2^/Z	*p*-Value
Age	54.3 ± 10.8	57.6 ± 10.4	2.244	0.026 *
Gender (Female, *n* (%))	75 (35.2%)	28 (39.4%)	0.411	0.521
BMI	23.8 ± 3.0	23.5 ± 2.5	−0.759	0.448
Duration of symptoms (months)	2.0 (1.0~5.0)	24.0 (12.0~36.0)	−11.357	<0.001 **
Smoking (*n* (%))	68 (31.9%)	19 (26.8%)	0.668	0.414
Drinking (*n* (%))	64 (30.0%)	16 (22.5%)	1.485	0.223
Number of lesion segments—single	60 (28.2%)	20 (28.2%)	0.000	1.000
≥2	153 (71.8%)	51 (71.8%)		
Surgical methods—Single ACDF	60 (28.2%)	20 (28.2%)	6.715	0.348
—Double ACDF	41 (19.3%)	15 (21.1%)		
—Three ACDF	12 (5.6%)	1 (1.4%)		
—ACCF	51 (23.9%)	12 (16.9%)		
—ACHDF	15 (7.0%)	10 (14.1%)		
—Laminoplasty	27 (12.7%)	11 (15.5%)		
—Laminectomy and fusion	7 (3.3%)	2 (2.8%)		
Surgical time (min)	124.8 ± 25.6	124.4 ± 24.7	−0.112	0.911
Blood loss (mL)	128.4 ± 76.0	128.5 ± 77.0	0.006	0.995
CCI	1.75 ± 1.6	2.1 ± 1.4	1.533	0.127
CCI classification—0–1 point	96 (45.1%)	26 (36.6%)	1.552	0.213
≥2 points	117 (54.9%)	45 (63.4%)		
CL	16.8 ± 9.9	14.1 ± 8.9	2.059	0.040 *
T1S	25.5 ± 7.6	24.4 ± 6.8	−1.027	0.305
C2-7SVA (mm)	15.9 ± 11.7	15.9 ± 10.3	0.007	0.994
CL (F)	−17.9 ± 9.8	−17.4 ± 8.6	0.401	0.689
T1S (F)	34.1 ± 9.2	34.1 ± 8.2	−0.043	0.965
C2-7SVA (F)	62.8 ± 13.6	63.3 ± 13.7	0.300	0.764
CL (E)	26.9 ± 12.1	27.9 ± 11.5	0.655	0.513
T1S (E)	22.1 ± 9.0	21.8 ± 8.2	−0.313	0.755
C2-7SVA (E)	2.2 (−11.1~7.8)	3.6 (−9.5~8.8)	−0.222	0.824
CL (ROM)	44.8 ± 14.0	45.4 ± 12.7	0.294	0.769
T1S (ROM)	−12.0 ± 10.3	−12.3 ± 9.0	−0.238	0.812
C2-7SVA (ROM)	−62.9 ± 22.0	−64.3 ± 20.8	−0.469	0.640
MSCC	0.42 ± 0.12	0.41 ± 0.14	−0.731	0.466
MCC	0.53 ± 0.13	0.52 ± 0.17	−0.319	0.750
TA	0.48 ± 0.13	0.48 ± 0.16	−0.109	0.913
TAR	0.44 ± 0.11	0.41 ± 0.15	−1.382	0.170
CR	0.23 ± 0.08	0.23 ± 0.09	−0.457	0.648
CCR	0.48 ± 0.13	0.46 ± 0.17	−0.868	0.387
Pre-VAS	4.3 ± 2.4	4.4 ± 2.2	0.117	0.907
Pre-NDI	16.6 ± 9.3	16.6 ± 8.3	0.008	0.994
Pre-mJOA	9.3 ± 1.3	9.8 ± 1.5	2.087	0.039 *

* and lighter gray shading signifies that *p* < 0.05. ** and dark gray shading signifies that *p* < 0.01.

**Table 4:** Using Binary Logistic Regression Analysis to Judge Independent Risk Factors.

	B	*p*-Value	OR	95% Confidence Interval of OR	
				Lower Bound	Upper Bound
Age	0.007	0.716	1.007	0.972	1.043
Duration of symptoms (M)	0.179	<0.001 **	1.196	1.135	1.261
CL	−0.025	0.194	0.975	0.939	1.013
Pre-mJOA	0.118	0.381	1.126	0.864	1.467

** and dark gray shading signifies that *p* < 0.05.

**Table 5:** Correlation between basic data and functional scores.

	**Pre-VAS**	**Pre-NDI**	**Pre-mJOA**	**Post-VAS** **(1)**	**Post-** **NDI** **(1)**	**Post-** **mJOA** **(1)**	**mJOA** **Recovery Rate% (1)**	**Post-** **VAS (3)**	**Post-** **NDI (3)**	**Post-** **mJOA** **(3)**	**mJOA** **Recovery Rate% (3)**	**Post-VAS (6)**	**Post-NDI (6)**	**Post-** **mJOA** **(6)**	**mJOA Recovery Rate% (6)**	**Post-** **VAS (12)**	**Post-** **NDI (12)**	**Post-** **mJOA** **(12)**	**mJOA Recovery** **Rate% (12)**	**Post-** **VAS (24)**	**Post-** **NDI (24)**	**Post-** **mJOA** **(24)**	**mJOA Recovery** **Rate% (24)**
Age	−0.147 *	−0.172 **	0.049	−0.116	−0.074	−0.129 *	−0.129 *	−0.056	−0.069	−0.127 *	−0.125 *	−0.072	−0.080	−0.129 *	−0.129 *	−0.105	−0.096	−0.122 *	−0.122 *	−0.102	−0.056	−0.144 *	−0.149 *
Gender	−0.076	−0.089	−0.074	−0.130 *	−0.119 *	0.044	0.064	−0.055	−0.081	0.032	0.052	−0.069	−0.034	0.039	0.060	−0.050	0.004	0.049	0.072	−0.023	−0.002	0.018	0.040
BMI	−0.004	−0.013	0.004	0.033	−0.015	−0.024	−0.032	−0.030	−0.027	−0.032	−0.038	−0.036	−0.038	−0.047	−0.055	−0.075	−0.063	−0.040	−0.049	−0.125 *	−0.109	−0.019	−0.032
Duration of symptoms	−0.061	−0.061	0.201 **	0.198 **	0.206 **	−0.567 **	−0.638 **	0.297 **	0.267 **	−0.557 **	−0.629 **	0.283 **	0.266 **	−0.559 **	−0.636 **	0.271 **	0.249 **	−0.560 **	−0.638 **	0.227 **	0.252 **	−0.552 **	−0.640 **
Smoking	−0.008	−0.028	−0.027	−0.042	−0.012	0.096	0.101	−0.039	−0.036	0.082	0.089	−0.057	−0.019	0.074	0.081	−0.034	0.004	0.083	0.093	−0.020	−0.006	0.076	0.081
Drinking	−0.064	−0.076	−0.010	−0.079	−0.060	0.053	0.055	−0.070	−0.087	0.047	0.050	−0.102	−0.090	0.045	0.050	−0.070	−0.053	0.047	0.054	−0.045	−0.061	0.023	0.027
lesion number	0.057	0.055	−0.144 *	0.042	0.091	−0.079	−0.029	0.018	0.110	−0.064	−0.014	0.016	0.075	−0.062	−0.009	−0.028	0.053	−0.072	−0.018	−0.063	0.089	−0.073	−0.020
CCI	−0.135 *	−0.119 *	0.011	−0.016	0.012	−0.032	−0.067	−0.006	0.055	−0.020	−0.054	−0.005	0.042	−0.021	−0.054	−0.021	0.019	−0.004	−0.038	0.023	0.088	−0.048	−0.082
CCI classification	−0.067	−0.062	0.029	−0.005	0.031	−0.035	−0.074	0.025	0.049	−0.019	−0.058	0.020	0.052	−0.025	−0.063	0.022	0.022	−0.011	−0.050	0.061	0.079	−0.037	−0.074
Pre-VAS	1	0.947 **	−0.010	0.604 **	0.605 **	−0.030	−0.024	0.476 **	0.46 9 **	−0.019	−0.013	0.402 **	0.406 **	−0.006	0.002	0.381 **	0.382 **	−0.015	−0.008	0.394 **	0.340 **	0.000	0.010
Pre-NDI		1	−0.026	0.597 **	0.627 **	−0.025	−0.020	0.464 **	0.498 **	−0.011	−0.007	0.396 **	0.433 **	0.000	0.007	0.370 **	0.406 **	−0.002	0.002	0.385 **	0.368 **	0.012	0.018
Pre-mJOA			1	0.022	−0.021	0.048	−0.199 **	0.045	0.031	0.051	−0.204 **	0.061	0.010	0.065	−0.196 **	0.056	−0.020	0.065	−0.197 **	0.026	0.015	0.097	−0.189 **
Correlation between Pre-operative Imaging Parameters and Functional Scores.																							
	**Pre-VAS**	**Pre-NDI**	**Pre-mJOA**	**Post-VAS** **(1)**	**Post-** **NDI** **(1)**	**Post-** **mJOA** **(1)**	**mJOA** **Recovery Rate%(1)**	**Post-** **VAS(3)**	**Post-** **NDI(3)**	**Post-** **mJOA** **(3)**	**mJOA** **Recovery Rate%(3)**	**Post-VAS(6)**	**Post-NDI(6)**	**Post-** **mJOA** **(6)**	**mJOA Recovery Rate%(6)**	**Post-** **VAS(12)**	**Post-** **NDI(12)**	**Post-** **mJOA** **(12)**	**mJOA Recovery** **Rate%(12)**	**Post-** **VAS(24)**	**Post-** **NDI(24)**	**Post-** **mJOA** **(24)**	**mJOA Recovery** **Rate%(24)**
CL	−0.184 **	−0.186 **	0.020	−0.094	−0.121 *	0.131 *	0.129 *	−0.079	−0.074	0.125 *	0.124 *	−0.056	−0.072	0.113	0.113	−0.048	−0.050	0.126 *	0.127 *	−0.018	−0.056	0.135 *	0.135 *
T1S	−0.100	−0.091	0.100	−0.120 *	−0.134 *	0.125 *	0.105	−0.062	−0.041	0.130 *	0.108	−0.017	−0.021	0.125 *	0.105	−0.019	−0.001	0.135 *	0.115	0.003	0.002	0.147 *	0.122 *
C2-7SVA	0.033	0.039	0.021	−0.031	−0.070	0.040	0.033	−0.021	−0.026	0.044	0.036	−0.003	0.022	0.049	0.040	−0.008	0.046	0.051	0.040	0.016	0.055	0.013	0.003
CL(F)	−0.155 **	−0.113	0.026	−0.118 *	−0.042	0.035	0.026	−0.072	−0.019	0.029	0.018	−0.045	−0.011	0.039	0.030	−0.034	0.022	0.054	0.045	−0.033	0.016	0.041	0.027
T1S(F)	−0.034	−0.012	0.022	−0.107	−0.057	0.045	0.065	−0.021	−0.006	0.063	0.081	−0.011	0.017	0.058	0.077	−0.012	0.023	0.060	0.080	0.014	0.030	0.081	0.096
C2-7SVA (F)	0.054	0.053	−0.040	0.003	−0.005	−0.028	−0.001	0.058	0.030	−0.014	0.013	0.052	0.082	−0.027	0.000	0.054	0.063	−0.024	0.003	0.051	0.046	−0.017	0.012
CL (E)	−0.051	−0.030	0.057	0.028	0.046	−0.025	−0.033	0.039	0.045	−0.027	−0.037	0.071	0.071	−0.042	−0.052	0.089	0.084	−0.026	−0.034	0.101	0.067	−0.007	−0.018
T1S (E)	−0.056	−0.042	0.058	−0.055	−0.064	0.089	0.065	−0.050	0.010	0.092	0.068	0.012	0.026	0.088	0.065	0.039	0.072	0.092	0.068	0.062	0.093	0.109	0.082
C2-7SVA (E)	0.002	0.012	−0.008	−0.036	−0.066	0.104	0.097	−0.070	−0.003	0.091	0.087	−0.069	−0.027	0.098	0.096	−0.057	0.002	0.098	0.093	−0.050	0.021	0.077	0.074
CL (ROM)	0.063	0.053	0.032	0.107	0.069	−0.046	−0.046	0.084	0.053	−0.044	−0.045	0.094	0.070	−0.064	−0.066	0.102	0.059	−0.061	−0.061	0.112	0.047	−0.035	−0.035
T1S (ROM)	−0.019	−0.026	0.032	0.048	−0.005	0.039	−0.001	−0.025	0.014	0.025	−0.013	0.021	0.007	0.025	−0.012	0.046	0.043	0.027	−0.012	0.042	0.056	0.024	−0.014
C2-7SVA (ROM)	−0.032	−0.026	0.020	−0.026	−0.041	0.087	0.065	−0.083	−0.020	0.069	0.050	−0.078	−0.069	0.082	0.064	−0.072	−0.038	0.081	0.060	−0.065	−0.015	0.062	0.042
MSCC	−0.008	−0.027	−0.621 **	−0.007	0.028	−0.031	0.121 *	−0.016	−0.019	−0.028	0.129 *	−0.070	−0.021	-0.040	0.122 *	−0.051	−0.027	−0.039	0.124 *	−0.037	−0.032	−0.061	0.117 *
MCC	−0.026	−0.035	−0.517 **	0.019	0.041	−0.045	0.091	0.015	0.003	−0.051	0.086	−0.038	−0.017	−0.059	0.084	−0.027	−0.018	−0.060	0.084	−0.022	−0.019	−0.084	0.073
TA	−0.009	0.001	0.714 **	−0.037	−0.068	0.150 *	−0.030	−0.071	−0.050	0.143 *	−0.042	−0.042	−0.064	0.147 *	−0.042	−0.053	−0.075	0.146 *	−0.044	−0.073	−0.050	0.176 **	−0.031
TAR	−0.028	−0.034	−0.852 **	−0.012	0.022	−0.082	0.137 *	−0.019	−0.024	−0.078	0.148 *	−0.049	−0.015	−0.088	0.143 *	−0.036	0.019	−0.085	0.148 *	−0.006	0.003	−0.123 *	0.130 *
CR	0.045	0.041	0.529 **	−0.003	−0.041	0.099	−0.029	−0.008	−0.009	0.098	−0.034	−0.005	−0.037	0.108	−0.029	0.015	−0.020	0.103	−0.037	0.004	−0.027	0.137 *	−0.015
CCR	−0.013	−0.038	−0.618 **	−0.039	−0.013	−0.037	0.125 *	−0.022	−0.051	−0.032	0.133 *	−0.062	−0.044	−0.047	0.125 *	−0.070	−0.052	−0.047	0.126 *	−0.068	−0.058	−0.072	0.117 *

* and lighter gray shading signifies that the correlation is significant at the 0.05 level (2-tailed). ** and dark gray shading signifies that the correlation is significant at the 0.01 level (2-tailed).

## Data Availability

Not applicable.
